# Supplementary material for: Roll-to-roll, high-resolution 3D printing of shape-specific particles
Source: Nature. 2024 Mar 13;627(8003):306–12. doi: 10.1038/s41586-024-07061-4 (PMC10937373; doi:10.1038/s41586-024-07061-4)
Supplement: Supplementary file 1 — Additional notes in support of the manuscript discussing the photochemistry of the process, film substrates, resin characterization, geometry and mouldability, interprint timing, optimization considerations and ceramics processing, and with respective additional references. [file 41586_2024_7061_MOESM1_ESM.pdf]

nature portfolio

https://doi.org/10.1038/s41586-024-07061-4

Supplementary information

Roll-to-roll, high-resolution 3D printing of shape-specific particles

In the format provided by the
authors and unedited

Supplementary Information for:

Roll-to-Roll, High-Resolution 3D Printing of Shape-Specific Particles

Jason M. Kronenfeld [1], Lukas Rother [2], Max A. Saccone [2,3], Maria T. Dulay [2], Joseph M. DeSimone [2,3]*

[1] Department of Chemistry, Stanford University, Stanford 94305, United States

[2] Department of Radiology, Stanford University, Stanford 94305, United States

[3] Department of Chemical Engineering, Stanford University, Stanford 94305, United States

* Corresponding author: jmdesimone@stanford.edu

Supplementary Information Table of Contents

Content	Description	*Page*
Table of Contents		*1*
Supplementary Note 1	Chemistry of the Printing Process	*2*
Supplementary Note 2	Film Substrates	*3 - 4*
Supplementary Table A	Material Comparison and Consideration for Usage in r2rCLIP System	*4*
Supplementary Note 3	Characterization of Resin and Analytical Technique	*5*
Supplementary Equation 1	Jacob's Equation	*5*
Supplementary Note 4	Particle Geometry Moldability Exploration	*6*
Supplementary Note 5	Inter-Print Manual Manipulation Timing	*7*
Supplementary Note 6	Parameter Optimization and Delamination	*8*
Supplementary Note 7	Polymer-Derived Ceramics Processing and Characterization	*9*
Supplementary References		*10 - 11*

Supplementary Note 1: Chemistry of the Printing Process

Within the CLIP system, there is a region of oxygen-inhibition through which light must pass. In this region, oxygen inhibition kinetics (formation of peroxy-radicals inhibiting initiation and radical polymerization propagation) dominate over radical polymerization. Thus, the resultant cure depth is the height of resin vertically cured from the region where the oxygen concentration in the liquid resin interface kinetically yields to radical initiators up to a region where accumulated light dosage is lower than E_{crit} (see *Extended Data Figure 2*). A resin formulation with a lower light penetration depth will result in better *z*-resolution but may in turn necessitate longer or higher cure dosages to achieve critical parameters. Extended exposure times are detrimental to the CLIP system as the dead zone will deplete over time during an exposure faster than oxygen diffusion to replenish the interface as would normally occur between exposures (referred to as “dark time”).

Few resin formulations exhibit parameters amenable to micron-thin cured layers; Kowsari et al. previously reported 4 μm lateral resolution utilizing an HDDA-HDDMA system [1]. Commercially available systems are tuned for commercial resolution ranges; the lowest penetration depth reported for a commercial resin is 42 - 53 μm for PR48 [2]. Overcoming these challenges in particle fabrication enables single-digit-micrometer voxel resolution. Addition of an UV absorbing compound can increase control over penetration depth but may also increase required exposure [3]. We found the HDDA-HDDMA resin presented herein was the easiest to work with because it has a low viscosity and dissolves well in isopropanol and acetone, while the cured resin does not swell in these solvents.

Supplementary Note 2: Film Substrates

We considered and assessed multiple film substrates on the r2rCLIP setup. The selection criteria included bending stiffness, chemical resistance, print adhesion, and commercial availability. If the bending stiffness of a film is too high, the film will not sit flat against the printhead, which will lead to uneven results and a polydisperse particle array. However, if the bending stiffness is too low, handling of the film will become more difficult as the force required for yielding and crazing is lower. The chemical resistance of the film is important, so it does not dissolve in the resin or the cleaning bath. Print adhesion describes how well the first printed resin layer sticks to the film substrate. If the first layer does not adhere, the print will fall off and fail. However, if the adhesion is too strong, it will be difficult to harvest particles from the film without breaking.

The following film substrates were tested: aluminum-coated PET film (48-2F-1M, CS Hyde), nickel (0.1*10, Moexsiac), aluminum (3A549, Grainger), acrylonitrile butadiene styrene (ABS; 8586K11, McMaster-Carr), fluorinated ethylene propylene (FEP) type A (23-1FEP-.5-50, CS Hyde), Kapton HN (18-2F-.5-50, CS Hyde), and skived polytetrafluoroethylene (PTFE; 15-1F-.5-100, CS Hyde; *Supplementary Table A*). The primary mechanism of polymeric component adherence to a film is secondary forces, intermolecular interactions between the polymer cured matrix and chosen substrate. The adherence of the cured resin might also be influenced by the irregularities on the surface of the substrate. These irregularities could include features like cracks, nicks, cavities, and other textural characteristics that the resin can mechanically latch onto. It is necessary to choose a substrate which exhibits a balance of these two factors. As macroscopic surface irregularities are detrimental to the final surface texture of a fabricated particle, we chose

to prioritize secondary-based interactions. Furthermore, more polar substrates are more amenable to soluble film coatings for alternative harvesting mechanisms.

Supplementary Table A: Material Comparison and Consideration for Usage in r2rCLIP System.

Material	Thickness (mm)	Bending stiffness	Chemical resistance	Print adhesion	Commercial source
Aluminum Coated PET	0.05	Good	Great	Great	CS Hyde
Nickel	0.1	Too high	Great	Great	Moexsiac
Aluminum	0.1	Too high	Great	Great	McMaster-Carr
ABS	0.3	Too high	Poor*	Good	McMaster-Carr
FEP	0.025	Too low	Great	Poor	CS Hyde
Kapton	0.025	Too low	Great	Poor	CS Hyde
Skived PTFE	0.025	Too low	Great	Poor	CS Hyde

* Film reacted with resin matrix and formed a thin, white film

Thickness reported as specified by the respective industrial products. Stiffness, resistance, and adhesion results are qualitative based on experience using the film in the setup and observations.

Several engineered controls maintain film regularity relative to the polymerization interface including micrometer precision adjustable screws and optical mechanical components. Platform tilt, film bulging, and window drumming lead to particle heterogeneity and were thus carefully designed against.

Supplementary Note 3: Characterization of Resin and Analytical Technique

As described in *Supplementary Equation 1* and *Extended Data Figure 2*, photopolymer matrix characteristic penetration depth and critical cure dosage both affect the finite cure depth of a voxel delivered dosage and must be carefully considered and tuned to resolve desired voxels without overcuring. This can be described by the Jacob's Equation [4],

$$C_d = D_p \ln\left(\frac{E_{max}}{E_{crit}}\right)$$ ***Supplementary Eq. 1***

where E_{max} is the light dosage delivered through the DMD as read through the In-Vision software, E_{crit} is the light dosage threshold to begin gelation, D_p is the penetration depth into the resin vat at which incident intensity is reduced by $1/e$, and C_d is the resulting vertical depth of resin cured within a given voxel. Smaller features are more susceptible to oligomer displacement and thus require a higher dosage (up to 5 x E_{crit}), however, higher or repeated dosages can accumulate oligomer formation leading to overcuring [4,5].

Previous experimental determination of working curve parameters has relied on exposing the resin atop a glass slide, or similar substrate, and measuring the resultant cured height via a variety of techniques from calipers to stylus profilometers [2]. However, we found that such an approach for a CLIP-based system assumes no polymerization-inhibition gradient. This results in an underestimation of dosage to cure as the light must first pass through a region of resin containing UV-absorber before interacting with the analytical region of interest at and above the polymerization interface. The glass slide introduces an additional medium, the glass slide, additionally affecting light penetration. Using a simple set of bridges with short overhang sections allows for analytical, local dosage determination.

Supplementary Note 4: Particle Geometry Moldability Exploration

Determination of particle geometry moldability is dependent on techniques available at the desired size range. Spherical geometries can be achieved by processes other than molding which are more readily applicable at scale and thus not considered [6]. General non-moldable features originate with consideration for undercuts, but several other factors must be considered. If a geometry may be created by one or more molds sections, the particle material must either be composed of thermoplastic such that the two components may be press-fit after molding or require an adhesion step. Parting line(s) will lead to anisotropy and act as a point of low yield stress. Alignment remains a challenge, especially if the mold component must be transferred before coupling, such as would be required for example to mold the demonstrated overhang clip shape. Complex molds may be more prone to variety of issues at the particle scale such as short shot – incomplete part formation due to insufficient or incomplete material filling the cavity. Some molding issues may still occur via other approaches including shrinkage, blisters, and bubbles. Molds requiring non-planar molds, mold inserts, vacuum, blow, or centrifuge molding may be difficult, expensive, or implausible at scale.

Supplementary Note 5: Inter-Print Manual Manipulation Timing

Manual manipulation between high-resolution print jobs was found to be equivalent to or greater than the amount of time required to fabricate an array of particles via r2rCLIP. To gain a baseline time for manual manipulation, we investigated the time between prints for all users (lab members) by parsing the log files from all high-resolution setups within the DeSimone research group. It is important to note that, in general, users are not rushing between a finished print and the start of the next print to minimize print downtime and other project print jobs can be up to orders of magnitude larger in size than particles given high-resolution CLIP versatility. Thus, statistics presented herein should be considered only as a baseline for comparison.

N = 6436 print jobs were analyzed out of n = 9527 print logs available by low and high pass filtering to remove timing associated with technical errors / testing, left to finish prints, etc. (inter-print range 5 - 600 seconds, intra-print range 20 - 60,000 seconds). We found that the average inter-print manual manipulation time was 4 ± 2 minutes (error presented as standard deviation of sampling), an order of magnitude higher than slowest inter-print r2rCLIP times.

Supplementary Note 6: Parameter Optimization and Delamination

As a wide variety of materials were employed on the r2rCLIP system, there inherently exists differences between the resin and polymeric properties of each system that must be optimized for compatibility with series components of the film system. While resin curing optimization is covered extensively within this manuscript, it should additionally be noted that further parameters must be considered and altered, for instance between fabricating a hydrogel and a pre-ceramic resin (or less to more viscous systems). For instance, high viscosity systems may require a longer dark time (time between exposures) to facilitate proper resin reflow. Additionally, delamination may be achieved more efficiently by heating the harvesting vat, such as was performed for the HDDA-HDDMA system during printing *en masse*. Larger surface area structures in the green state may be more prone to cracking when sonication is utilized during the delamination step, however, introducing a secondary-cure post-wash qualitatively decreases the likelihood of cracking. Optimization of curing parameters under inert atmosphere or elevated temperatures, as previously shown [7–9], additionally decreases the likelihood of particle failure during harvesting and further addresses desired mechanical properties.

Supplementary Note 7: Polymer-Derived Ceramics Processing and Characterization

To print technical ceramic particles, we explored various pre-ceramic polymer resins. Here, we report characterization of Carbon SIL30 resin, a commercially available silicone-based 3D printing resin. *Extended Data Figure 3a* shows that the mass yield of this resin is 9.5% after heating to 800 ˚C in a nitrogen atmosphere. Samples were 3D printed and then pyrolyzed at 800 ˚C, yielding a black material which was crushed for XRD analysis, then subsequently annealed at 1200 ˚C and 1400 ˚C. *Extended Data Figure 3b* shows the XRD patterns of these samples; the samples annealed at 800 ˚C and 1200 ˚C remain amorphous, while the sample annealed at 1400 ˚C has reflections indicating the presence of crystalline α-Si_3N_4, a technical ceramic with desirable mechanical properties. To print reliably with higher resolution, the ceramic particle geometries in *Figure 4a,b*, were printed using the custom HDDA-HDDMA resin developed in this work (as described in Methods) combined with a component of SIL30 resin. This demonstrates a clear path towards printing of microscale technical ceramic silicon nitride particles with arbitrary shapes.

Supplementary References

1. Kowsari, K. *et al.* Photopolymer formulation to minimize feature size, surface roughness, and stair-stepping in digital light processing-based three-dimensional printing. *Addit. Manuf.* **24**, 627–638 (2018).
2. Bennett, J. Measuring UV curing parameters of commercial photopolymers used in additive manufacturing. *Addit. Manuf.* **18**, 203–212 (2017).
3. Bail, R., Hong, J. Y. & Chin, B. D. Effect of a red-shifted benzotriazole UV absorber on curing depth and kinetics in visible light initiated photopolymer resins for 3D printing. *J. Ind. Eng. Chem.* **38**, 141–145 (2016).
4. Jacobs, P. F. *Fundamentals of Stereolithography*. (Society of Manufacturing Engineers, 1992).
5. Pritchard, Z. D., de Beer, M. P., Whelan, R. J., Scott, T. F. & Burns, M. A. Modeling and Correcting Cure-Through in Continuous Stereolithographic 3D Printing. *Adv. Mater. Technol.* **4**, 1900700 (2019).
6. Stanisz, M., Klapiszewski, Ł. & Jesionowski, T. Spherical Particle Technology and Engineering: Fabrication and Practical Utility. in *Practical Aspects of Chemical Engineering* (eds. Ochowiak, M., Woziwodzki, S., Mitkowski, P. T. & Doligalski, M.) 430–440 (Springer International Publishing, 2020). doi:10.1007/978-3-030-39867-5_45.
7. Uzcategui, A. C., Muralidharan, A., Ferguson, V. L., Bryant, S. J. & McLeod, R. R. Understanding and Improving Mechanical Properties in 3D printed Parts Using a Dual-Cure Acrylate-Based Resin for Stereolithography. *Adv. Eng. Mater.* **20**, 1800876 (2018).
8. Riccio, C. *et al.* Effects of Curing on Photosensitive Resins in SLA Additive Manufacturing. *Appl. Mech.* **2**, 942–955 (2021).

9. Wada, J. *et al.* Effect of Nitrogen Gas Post-Curing and Printer Type on the Mechanical Properties of 3D-Printed Hard Occlusal Splint Material. *Polymers* **14**, 3971 (2022).
